# Kaposi’s sarcoma in people living with HIV/aids in a public referral hospital in Peru

**DOI:** 10.17843/rpmesp.2022.393.10883

**Published:** 2022-09-30

**Authors:** Lucía Maryelena Mendoza Mori, Jim Brian Valenzuela Medina, José Eduardo Gotuzzo Herencia, Francisco Gerardo Bravo Puccio, Fernando Alonso Mejía Cordero, Salim Mohanna Barrenechea, Elsa Violeta González Lagos

**Affiliations:** 1 “Alexander von Humboldt” Institute of Tropical Medicine, Lima, Peru. “Alexander von Humboldt” Institute of Tropical Medicine Lima Peru; 2 Universidad Peruana Cayetano Heredia, Lima, Peru. Universidad Peruana Cayetano Heredia Universidad Peruana Cayetano Heredia Lima Peru; 3 Dermatology and pathology service, Hospital Cayetano Heredia, Lima, Peru Dermatology and pathology service Hospital Cayetano Heredia Lima Peru; 4 Universidad Nacional Mayor de San Marcos, Lima, Peru. Universidad Nacional Mayor de San Marcos Universidad Nacional Mayor de San Marcos Lima Peru

**Keywords:** Kaposi’s Sarcoma, HIV, AIDS, Latin America

## Abstract

Kaposi’s sarcoma (KS) is the most frequent cancer in people living with HIV. Research on this condition is scarce in the region, therefore, this article aimed to describe the demographic, clinical and therapeutic characteristics of patients with HIV who developed KS at the Cayetano Heredia Hospital between 2000 and 2018. A total of 129 KS cases were identified, with a median age of 33 years, predominantly males with 92% (119/129), and mostly men who have sex with men (MSM). The median time from HIV diagnosis to KS diagnosis was five months, associated with a CD4 lymphocyte count of 64 cells/μL (IQR: 33-185) at KS diagnosis. Cutaneous involvement was the most common presentation; however, at least half also had the visceral form.

## INTRODUCTION

Kaposi’s sarcoma (KS) is the most common cancer in people living with HIV (PLWHA) in developing countries ^1^. Antiretroviral therapy (ART) has successfully reduced the incidence of KS worldwide ^2^ ^,^ ^3^; however, the risk of KS remains high in resource-poor countries, where treatment is still not universally available or free of charge.

Human herpesvirus 8 (HHV-8) is the etiologic agent of KS, and is a necessary but not sufficient cause for developing this disease ^4^. Seroprevalence varies geographically between continents, being higher in regions such as Africa and Latin America ^5^ and less prevalent in Europe and North America ^4^. It is also highly prevalent among men who have sex with men (MSM), particularly those with HIV co-infection ^6^ ^,^ ^7^.

In Peru, ART has been administered since 2004, when the Ministry of Health (MINSA) approved the ruling for its free distribution in public hospitals. Since then, as in the rest of the world, the impact has been decisive in reducing the number of cases of opportunistic infections and HIV-related neoplasms. In 2006, Mohanna *et al*. reported that the incidence of epidemic KS was 20.26 per 1000 HIV patients treated between 1987 and 2003, with a male:female ratio of 15:1 ^8^.

This study aimed to describe the demographic, clinical, laboratory and therapeutic characteristics of patients with HIV who developed KS over a period of time, as well as to evaluate the characteristics of cases who developed KS, regardless of CD4 lymphocyte count.

**KEY MESSAGES**

Motivation for the study: Kaposi’s sarcoma (KS) is the most frequent type of cancer in our setting, so there is a need to describe the epidemiological and clinical characteristics of our patients.

Main findings: KS is more frequent in young male homosexuals with low CD4 lymphocyte count and diagnosed less than one year after HIV diagnosis.

Implications: antiretroviral treatment should be provided in a timely manner to reduce the risk of any type of cancer or opportunistic infections.

## THE STUDY

### Study design and population

We carried out a descriptive study, case series type, taking information from the records of an HIV/AIDS cohort (COVIHS) of the Cayetano Heredia Hospital. This cohort aimed to collect information on the demographic, clinical and laboratory characteristics of PLWHA treated at the hospital. We included in our study all PLWHA over 18 years of age who developed KS between January 1, 2000 and December 31, 2018 who were enrolled in the cohort.

### Definition of variables

Demographic characteristics included age at KS diagnosis, sex and sexual orientation (heterosexual or MSM). The clinical characteristics included organ involvement which was classified as cutaneous, visceral or both, and was presented by using frequencies. The time elapsed from HIV diagnosis to KS was divided into three periods: 0 to 2 months, 2 to 12 months or more than 12 months. The laboratory values included CD4 lymphocyte count (<200 cells/µL, 200-299 cells/µL or ≥300 cells/µL) and the Log10 viral load (<2.7; 2.7-3.9; 4-4.9 or ≥5 copies/mL), both based on the most recent result registered between six months before and one month after the date of KS diagnosis ^4^. Treatment included ART and chemotherapy. Missing data were classified in the unknown group. Age, time from HIV diagnosis to KS, CD4 lymphocyte count, and HIV RNA level were also reported as median plus interquartile range (IQR). The number of cases per calendar year was also presented.

### Data Management

The information was obtained by authorized extraction from the COVIHS database. It should be noted that, in order to complete the missing data for this cohort, it was necessary to review the medical records from the hospital archives service. However, for deceased participants, some variables could not be completed because the medical record had already been discarded. To guarantee confidentiality, personal information (names, surnames and identity number) was eliminated and restricted for new users. To reduce the probability of systematic errors, we verified duplicate identification codes and inconsistent data. Finally, the variables were entered into a Excel database, where we determined the frequencies of each variable and proceeded to the statistical analysis.

### Statistical analysis

Data were analyzed with STATA software, version 14. Continuous variables were summarized with median and interquartile range (IQR) and categorical variables were summarized with percentages and absolute values.

### Ethical considerations

The study protocol was approved by the Institutional Ethics Committee of the Cayetano Heredia University (E074-14-20). Informed consent was not required because the information was collected from the database of a previous cohort. Data collection and management was carried out within the standards of care, guaranteeing the confidentiality of personal information.

## FINDINGS

We identified 129 KS cases between 2000 and 2018. The median age at diagnosis was 33 years (IQR: 28-41). Most were male (92.3%, 119/129) and of these, more than two-thirds (88/129) were reported as MSM, compared to all women who were reported as heterosexual. The median time from HIV diagnosis to KS diagnosis was 4 months, most (65.9%, 85/129) were diagnosed during the first year, after HIV diagnosis. The median CD4 lymphocyte count was 64 cells/µL (IQR, 33-185); however, 17.8% (23/129) had more than 200 cells/µL at KS diagnosis. The demographic and clinical characteristics of the patients are summarized in Table 1.

**Table 1:** Characteristics of the cases at the time of diagnosis of Kaposi's sarcoma (n=129).

Characteristics	n (%)
Age	
Median (IQR)	33 (28-41)
Sex	
Male	119 (92.3)
Female	10 (7.7)
Sexual orientation	
MSM	88 (68.2)
Heterosexual men	29 (22.5)
Heterosexual women	10 (7.8)
Unknown	2 (1.5)
Involvement	
Cutaneous	55 (42.6)
Cutaneous and visceral	49 (38.0)
Visceral	25 (19.4)
Time from HIV diagnosis to KS (months)	
Median (IQR)	4 (1-20)
0-2 months	48 (37.2)
2-12 months	37 (28.7)
>12 months	44 (34.1)
CD4 lymphocyte count (cells/μL)	
Median (IQR)	64 (35-185)
< 200	94 (72.9)
200 - 299	15 (11.6)
≥300	8 (6.2)
Unknown	12 (9.3)
Log10 viral load (copies/mL)	
Median (IQR)	5.1 (3.9-5.7)
<2.7	15 (11.6)
2.7-3.9	14 (10.9)
4-4.9	24 (18.6)
≥ 5	59 (45.7)
Unknown	17 (13.2)
KS treatment	
ART	42 (32.6)
Chemotherapy	
Paclitaxel	59 (45.7)
Other	11 (8.5)
Unknown	17 (13.2)
Number of cases per calendar year	
2000	1 (0.8)
2001	1 (0.8
2002	0 (0)
2003	3 (2.3)
2004	1 (0.8)
2005	2 (1.6)
2006	1 (0.8)
2007	1 (0.8)
2008	8 (6.2)
2009	9 (6.9)
2010	9 (6.9)
2011	7 (5.4)
2012	6 (4.6)
2013	13 (10.1)
2014	12 (9.3)
2015	12 (9.3)
2016	14 (10.9)
2017	15 (11.6)
2018	14 (10.9)

IQR: interquartile range; KS: Kaposi´s sarcoma; MSM: men who have sex with men; ART: antiretroviral therapy.

Cutaneous involvement was the most common presentation in 80.6% (104/129) of the patients, and of these, 38.0% (49/129) also developed the visceral form. Regarding visceral involvement, the gastrointestinal system was the most affected (Table 2). On the other hand, more than half of the patients received chemotherapy, with paclitaxel being the most commonly used drug. ART as the only treatment was received by one third of the patients. There was an increase in the number of KS cases per calendar year, due to the improvement of the data collection system and the fact that more patients were enrolled in the COVIHS each year.

**Table 2:** Visceral involvement according to the affected organ. The number of cases with visceral involvement and the frequency of the most affected systems are summarized (n=74).

Visceral involvement	n (%)
GIT	53 (71.6)
Lymph nodes	7 (9.5)
GIT + Lymph nodes	7 (9.5)
Lungs	2 (2.7)
GIT + lungs	3 (4.1)
Lymph nodes + lungs	1 (1.3)
GIT + Lymph nodes + lungs	1 (1.3)

GIT: Gastrointestinal tract

Table 3 describes the information of the eight cases that presented CD4 lymphocyte counts above 300 cells/µL at the time of KS diagnosis. The median age was 36.5 years (IQR: 29-41.5); there was only one female and all males reported their sexual orientation as MSM. The median time from HIV diagnosis to KS diagnosis was 27.1 months (IQR: 5.7-104.2); the median CD4 lymphocyte count was 375 cells/µL (IQR: 312-489.5) and the median Log10 viral load was 2.1 copies/ml (IQR: 1.5-4.8). Skin and visceral disease were reported in 5 and 3 cases, respectively. Only six patients were being treated with ART prior to KS diagnosis and five received chemotherapy as targeted therapy for KS.

**Table 3:** Characteristics of Kaposis sarcoma cases with CD4 ≥ 300 cells/μL at diagnosis (n=8).

	Sex	Age	Sexual orientation	HIV to KS ^a^	CD4	Viral load	Type	ART	ART scheme	KS treatment
1	M	39	MSM	14 months	312	400	Cutaneous	Yes	NNRTI	Unknown
2	M	28	MSM	3 years	323	UD	Cutaneous	Yes	NNRTI	CT
3	M	40	MSM	9 years	312	UD	Cutaneous	Yes	PI	ART
4	M	32	MSM	3 months	427	UD	Cutaneous	Yes	NNRTI	CT
5	M	42	MSM	5 months	479	234	Cutaneous	Yes	NNRTI	ART
6	F	48	MSM	18 years	614	UD	Visceral	Yes	NNRTI	CT
7	M	34	Heterosexual	6 years	302	113805	Visceral	No	Unknown	CT
8	M	27	MSM	5 months	493	92066	Visceral	No	Unknown	CT

a Time elapsed from HIV diagnosis to Kaposi’s sarcoma diagnosis. SK: Kaposi’s sarcoma; M: male; F: female; MSM: men who have sex with men; UD: undetectable; ART: antiretroviral therapy; NNRTI: non-nucleoside reverse transcriptase inhibitors; PI: protease inhibitors; CT: chemotherapy.

## DISCUSSION

KS remains the most frequent type of cancer in low- and middle-income settings ^9^. Our results are comparable with those published worldwide ^10^. Among the main findings, we highlight that this neoplasm is predominantly found in young males, with sexual orientation toward MSM, with CD4 lymphocyte counts below 100, detectable viral load, and with visceral presentation in more than 50% of cases.

Bohlius *et al*. conducted a multiregional cohort study in all five continents. They found that, in three of the four regions, men were the largest group, 94% in Latin America, 93% in North America and 90% in Europe ^4^, which is similar to our findings; however, in South Africa, women were the more affected than men, probably due to the higher female prevalence of HIV in that continent. On the other hand, a much higher median age at KS diagnosis was found in North America and Europe, 42.9 years (IQR: 37.6-48.7) and 40.2 years (IQR: 34.1-47.8), respectively ^4^. Also, in these regions, MSM were found to be at higher risk of developing KS, in contrast to heterosexual men or women, whose frequency ranged from 65% to 75% for the Americas and Europe, probably due to the high rates of co-infection with HVH-8 among MSM ^11^. Our study describes a median CD4 lymphocyte count at KS diagnosis of 64 cells/µL (IQR: 33-185), which is comparable to that described in other continents; however, a higher median is described in higher income countries, such as North America and Europe, 87 cells/µL (IQR: 20-280), 180 cells/µL (IQR: 60-348), respectively ^4^. 

Although ART has brought great benefits, KS is now associated with higher CD4 lymphocyte count levels, even in treatment-adherent patients ^12^ ^,^ ^13^. Studies have documented this finding on CD4 lymphocyte count trends since Maurer *et al*. reported a group of nine HIV patients on ART who developed KS in the context of good immune status (CD4 greater than 300 cells/µL) and undetectable viral load ^14^.

Accordingly, our study found eight individuals with CD4 lymphocyte counts above 300 cells/µL and, of these, six with undetectable viral load. Some authors have hypothesized that the high number of elderly patients might alter the integrity of the immune system in long-term HIV infection ^15^. However, Unemori *et al*. in 2013, explored the possible mechanisms of this phenomenon and found that progressive loss of immune function during advanced age, referred to as immunosenescence, could be directly associated with the development of KS ^16^.

The main limitation of this study was information bias, because of the retrospective design over a period of 18 years. The information was limited to what was available in a database and to what was additionally obtained from the review of medical records of patients who were still alive. Another limitation was selection bias, since all patients with HIV who developed KS were selected. However, the actual number of cases was underestimated, due to the lack of data collection techniques in the past, so the prevalence or incidence of this disease could not be determined.

In conclusion, the characteristics associated with KS found in our setting were similar to those described in the literature. It is predominantly found in young males with sexual orientation towards MSM, with low CD4 lymphocyte counts. Cutaneous involvement was the most frequent presentation, and at least half had associated visceral involvement. It is also important to highlight that, in this series, as in others described recently, patients with KS with CD4 lymphocyte counts above 300 cells/µL were also identified.
