# Supplementary material for: Kaposi’s sarcoma in people living with HIV/aids in a public referral hospital in Peru
Source: Rev Peru Med Exp Salud Publica. 2022 Sep 30;39(3):352–6. doi: 10.17843/rpmesp.2022.393.10883 (PMC11397678; doi:10.17843/rpmesp.2022.393.10883)
Supplement: Translation

# Translation: Sarcoma de Kaposi en personas que viven con VIH-SIDA en un hospital público de referencia en Perú

**Authors:** Lucía Maryelena Mendoza Mori, Jim Brian Valenzuela Medina, José Eduardo Gotuzzo Herencia, Francisco Gerardo Bravo Puccio, Fernando Alonso Mejía Cordero, Salim Mohanna Barrenechea, Elsa Violeta González Lagos

**Affiliations:** 1 Instituto de Medicina Tropical «Alexander von Humboldt», Lima, Perú.; 2 Universidad Peruana Cayetano Heredia, Lima, Perú.; 3 Servicio de dermatología y patología, Hospital Cayetano Heredia, Lima, Perú.; 4 Universidad Nacional Mayor de San Marcos, Lima, Perú.

**Keywords:** Sarcoma de Kaposi, VIH, SIDA, América Latina

## Abstract

El sarcoma de Kaposi (SK) es el cáncer más frecuente en las personas que viven con VIH. Las investigaciones sobre esta condición son escasas en la región, por lo que, el objetivo de este artículo fue describir las características demográficas, clínicas y terapéuticas de los pacientes con VIH que desarrollaron SK en el Hospital Cayetano Heredia entre el 2000 y 2018. Se identificaron 129 casos de SK, con una mediana de edad de 33 años, con predominio en varones con el 92% (119/129), y en su mayoría hombres que tienen sexo con hombres (HSH). La mediana de tiempo desde el diagnóstico de VIH hasta el del SK fue de cinco meses, asociado con un recuento de linfocitos CD4 de 64 células/µL (RIC: 33-185) al momento del diagnóstico de SK. El compromiso cutáneo fue el más común; sin embargo, al menos la mitad de ellos también tuvo la forma visceral.

## INTRODUCCIÓN

El sarcoma de Kaposi (SK) es el cáncer más frecuente en personas que viven con VIH (PVV) en los países en desarrollo ^1^. El tratamiento antirretroviral (TARV) ha logrado reducir con éxito la incidencia del SK en todo el mundo ^2^ ^,^ ^3^; sin embargo, el riesgo de este tipo de cáncer permanece alto en los países de escasos recursos, donde el tratamiento todavía no tiene cobertura universal ni gratuita. 

El agente etiológico del SK es el herpes virus humano 8 (HVH-8), una causa necesaria, pero no suficiente para desarrollar esta enfermedad ^4^. La seroprevalencia varía geográficamente entre continentes, siendo más alta en regiones como África y América Latina ^5^ y menos prevalente en Europa y Norteamérica ^4^. Asimismo, presenta una alta prevalencia entre los hombres que tienen sexo con hombres (HSH), particularmente en aquellos con coinfección por VIH ^6^ ^,^ ^7^.

En Perú, el TARV se administra desde el 2004, cuando el Ministerio de Salud (MINSA) aprobó el dictamen para su distribución gratuita en los hospitales públicos. Desde entonces, y como en el resto del mundo, el impacto ha sido determinante en la disminución del número de casos de infecciones oportunistas y neoplasias relacionadas con el VIH. En el 2006, Mohanna *et al*. reportaron que la incidencia del SK epidémico era de 20,26 por 1000 pacientes con VIH, atendidos entre 1987 y 2003, con una relación hombre: mujer de 15:1^8^.

Este estudio tuvo como objetivo describir las características demográficas, clínicas, laboratoriales y terapéuticas de los pacientes con VIH que desarrollaron SK en un periodo de tiempo, así como evaluar las características de los casos que desarrollaron SK, independientemente del recuento de linfocitos CD4.

**MENSAJE CLAVE**

Motivación para realizar el estudio: el sarcoma de Kaposi (SK) es el tipo de cáncer más frecuente en nuestro medio, por lo que existe la necesidad de describir las características epidemiológicas y clínicas de nuestros pacientes.

Principales hallazgos: el SK es más frecuente en homosexuales varones jóvenes, con recuento de linfocitos CD4 bajo y con presentación diagnóstica menor de un año desde el diagnóstico de VIH.

Implicancias: se debe brindar oportunamente el tratamiento antirretroviral para reducir el riesgo de cualquier tipo de cáncer o de infecciones oportunistas.

## EL ESTUDIO

### Diseño y población de estudio

Se realizó un estudio descriptivo, tipo serie de casos, que tomó la información de registros de una cohorte de VIH/SIDA (COVIHS) del Hospital Cayetano Heredia. Esta cohorte busca recolectar información sobre las características demográficas, clínicas y laboratoriales de las PVV que se atienden en dicho hospital. Se incluyeron a todas las PVV mayores de 18 años que desarrollaron SK entre el 1 de enero de 2000 y el 31 de diciembre de 2018, y que estuvieron enroladas en dicha cohorte. 

### Definición de variables

Las características demográficas incluyeron la edad al momento del diagnóstico de SK, el sexo y la orientación sexual (heterosexual u HSH). Dentro de las características clínicas, el compromiso se clasificó en cutáneo, visceral o ambos, y se presentó con frecuencias según órgano comprometido. El tiempo transcurrido desde el diagnóstico de VIH hasta el SK se dividió en tres períodos: de 0 a 2 meses, de 2 a 12 meses o mayor de 12 meses. Los valores de laboratorio fueron: el recuento de linfocitos CD4 (<200 células/µL, 200-299 células/µL o ≥300 células/µL) y el Log10 de la carga viral (<2,7; 2,7-3,9; 4-4,9 o ≥5 copias/mL), ambos basados en el resultado más reciente registrado entre seis meses antes y un mes después de la fecha del diagnóstico de SK ^4^. El tratamiento incluyó al TARV y la quimioterapia. Los datos faltantes se clasificaron en el grupo desconocido. La edad, el tiempo desde el diagnóstico del VIH hasta el SK, el recuento de linfocitos CD4 y el nivel de ARN del VIH también se informaron como mediana más rango intercuartílico (RIC). También se presentó el número de casos por año calendario. 

### Manejo de datos

La información se obtuvo mediante la extracción autorizada de la base de datos de la COVIHS. Cabe resaltar que, para completar los datos faltantes de dicha cohorte, fue necesaria la revisión de las historias clínicas en el servicio de archivos del hospital. Sin embargo, para los participantes fallecidos, no se pudo completar algunas variables, debido a que la historia clínica ya había sido descartada. Para garantizar la confidencialidad, la información personal (nombres, apellidos y número de identidad) fue eliminada, restringiéndose en nuevos usuarios. Para reducir la probabilidad de cometer errores sistemáticos se verificaron los códigos de identificación duplicados y datos inconsistentes. Finalmente, se tabularon las variables en una nueva base de datos de Excel, donde se determinaron las frecuencias de cada una y se procedió al análisis estadístico. 

### Análisis estadístico

Los datos se analizaron con el *software* STATA, versión 14. Las variables continuas se resumieron con la mediana y el rango intercuartilico (RIC) y las variables categóricas se resumieron con porcentajes y valores absolutos.

### Consideraciones éticas

El protocolo del estudio fue aprobado por el Comité Institucional de Ética de la Universidad Cayetano Heredia (E074-14-20). Se exoneró el uso de consentimiento informado, ya que la información recopilada provino de una base de datos perteneciente a una cohorte. La recolección y el manejo de los datos se realizó dentro de los estándares de cuidado, garantizando la confidencialidad de la información personal.

## HALLAZGOS

Entre el 2000 y 2018 se identificaron 129 casos de SK. La mediana de edad al momento del diagnóstico fue de 33 años (RIC: 28-41). Fueron predominantemente hombres en el 92,3% (119/129) y entre ellos, más de dos tercios (88/129) se reportaron como HSH, en comparación con todas las mujeres que se reportaron como heterosexuales. La mediana de tiempo desde el diagnóstico de VIH hasta el diagnóstico de SK fue de 4 meses, siendo el 65,9% (85/129) diagnosticados durante el primer año, después del diagnóstico de VIH. La mediana del recuento de linfocitos CD4 fue de 64 células/µL (RIC, 33-185); sin embargo, alrededor del 17,8% (23/129) presentaron más de 200 células/µL al momento del diagnóstico de SK. Las características demográficas y clínicas de los pacientes se resumen en la Tabla 1.

**Tabla 1:** Características de los casos al momento del diagnóstico de sarcoma de Kaposi (n=129).

Características	n (%)
Edad	
Mediana (RIC)	33 (28-41)
Sexo	
Masculino	119 (92,3)
Femenino	10 (7,7)
Orientación sexual	
HSH	88 (68,2)
Hombres heterosexuales	29 (22,5)
Mujeres heterosexuales	10 (7,8)
Desconocido	2 (1,5)
Compromiso	
Cutáneo	55 (42,6)
Cutáneo y visceral	49 (38,0)
Visceral	25 (19,4)
Tiempo desde el diagnóstico de VIH hasta el SK (meses)	
Mediana (RIC)	4 (1-20)
0-2 meses	48 (37,2)
2-12 meses	37 (28,7)
>12 meses	44 (34,1)
Recuento de linfocitos CD4 (células/µL)	
Mediana (RIC)	64 (35-185)
< 200	94 (72,9)
200 - 299	15 (11,6)
≥300	8 (6,2)
Desconocido	12 (9,3)
Log10 carga viral (copias/mL)	
Mediana (RIC)	5.1 (3,9-5,7)
<2.7	15 (11,6)
2.7-3.9	14 (10,9)
4-4.9	24 (18,6)
≥ 5	59 (45,7)
Desconocido	17 (13,2)
Tratamiento para el SK	
TARV	42 (32,6)
Quimioterapia	
Paclitaxel	59 (45,7)
Otros	11 (8,5)
Desconocido	17 (13,2)
Número de casos por año calendario	
2000	1 (0,8)
2001	1 (0,8)
2002	0 (0)
2003	3 (2,3)
2004	1 (0,8)
2005	2 (1,6)
2006	1 (0,8)
2007	1 (0,8)
2008	8 (6,2)
2009	9 (6,9)
2010	9 (6,9)
2011	7 (5,4)
2012	6 (4,6)
2013	13 (10,1)
2014	12 (9,3)
2015	12 (9,3)
2016	14 (10,9)
2017	15 (11,6)
2018	14 (10,9)

RIC: rango intercuartílico; SK: sarcoma de Kaposi; HSH: hombres que tienen sexo con hombres; TARV: terapia antirretroviral.

El compromiso cutáneo fue la presentación más común con el 80,6% (104/129), y de ellos, el 38,0% (49/129) también desarrollaron la forma visceral. Con relación al compromiso visceral, el sistema gastrointestinal fue el más afectado (Tabla 2). Por otro lado, más de la mitad de los pacientes recibieron quimioterapia, siendo el paclitaxel, el fármaco más utilizado. El TARV como único tratamiento fue recibido por la tercera parte de los pacientes. Se observo un incremento de casos de SK por año calendario, debido a que se mejoró el sistema de recolección de datos y a que cada año se enrolaban más pacientes a la COVIHS. 

**Tabla 2:** Compromiso visceral según órgano afectado. Se resume el número de casos que presentaron compromiso visceral y la frecuencia de los sistemas más afectados (n=74).

Compromiso visceral	n (%)
TGI	53 (71,6)
Ganglios linfáticos	7 (9,5)
TGI + ganglios linfáticos	7 (9,5)
Pulmones	2 (2,7)
TGI + pulmones	3 (4,1)
Ganglios linfáticos + pulmones	1 (1,3)
TGI + ganglios linfáticos + pulmones	1 (1,3)

TGI: tracto gastrointestinal

La Tabla 3 describe la información de los ocho casos que presentaron recuentos de linfocitos CD4 superiores a 300 células/µL al momento del diagnóstico de SK. La mediana de edad fue de 36,5 años (RIC: 29-41,5); solo hubo una mujer y todos los hombres declararon su orientación sexual como HSH. La mediana del tiempo desde el diagnóstico del VIH hasta el diagnóstico de SK fue de 27,1 meses (RIC: 5,7-104,2); la mediana del recuento de linfocitos CD4 fue de 375 células/µL (RIC: 312-489,5) y la mediana del Log10 de la carga viral fue de 2,1 copias/ml (RIC: 1,5-4,8). Las enfermedades cutáneas y viscerales se notificaron en 5 y 3 casos, respectivamente. Solo seis pacientes estaban siendo tratados con TARV antes del diagnóstico de SK y cinco recibieron quimioterapia como tratamiento específico para el SK.

**Tabla 3:** Características de los casos de sarcoma de Kaposi con CD4 ≥ 300 células/μL en el momento del diagnóstico (n=8).

	Sexo	Edad	Orientación sexual	VIH al SK^a^	CD4	Carga viral	Forma	TARV	Esquema TARV	Tratamiento SK
1	M	39	HSH	14 meses	312	400	Cutánea	Sí	INNTR	Desconocido
2	M	28	HSH	3 años	323	ID	Cutánea	Sí	INNTR	QT
3	M	40	HSH	9 años	312	ID	Cutánea	Sí	IP	TARV
4	M	32	HSH	3 meses	427	ID	Cutánea	Sí	INNTR	QT
5	M	42	HSH	5 meses	479	234	Cutánea	Sí	INNTR	TARV
6	F	48	Heterosexual	18 años	614	ID	Visceral	Sí	INNTR	QT
7	M	34	HSH	6 años	302	113805	Visceral	No	Desconocido	QT
8	M	27	HSH	5 meses	493	92066	Visceral	No	Desconocido	QT

a Tiempo transcurrido desde el diagnostico de VIH al diagnostico de sarcoma de Kaposi.

SK: sarcoma de Kaposi; M: masculino; F: femenino; HSH: hombres que tienen sexo con hombres; ID: indetectable; TARV: terapia antirretroviral; INNTR: inhibidores no nucleósidos de la transcriptasa reversa; IP: inhibidores de proteasa; QT: quimioterapia.

## DISCUSIÓN

El SK sigue siendo el tipo de cáncer más frecuente en entornos de ingresos bajos y medios ^9^. Los resultados del presente estudio son comparables con lo descrito en la literatura mundial ^10^. Entre los principales hallazgos se destaca que esta neoplasia es predominante de varones jóvenes, con orientación sexual hacia HSH, con recuentos de linfocitos CD4 por debajo de 100, carga viral detectable, y con forma de presentación visceral en más del 50% de casos. 

Bohlius *et al*. llevaron a cabo un estudio tipo cohorte multirregional en los cinco continentes. Encontraron que, en tres de las cuatro regiones, los hombres fueron el grupo más grande, 94% en América Latina, 93% en Norteamérica y 90% en Europa ^4^ similar a nuestros hallazgos; sin embargo, en Sudáfrica, las mujeres fueron el sexo más afectado, probablemente debido a la mayor prevalencia femenina del VIH en ese continente. Por otro lado, en Norteamérica y Europa, se encontró una mediana de edad mucho más alta al momento del diagnóstico del SK, esto es 42,9 años (RIC: 37,6-48,7) y 40,2 años (RIC: 34,1-47,8), respectivamente ^4^. Asimismo, en dichas regiones se notó que los HSH tuvieron más riesgo de desarrollar SK, a diferencia de los varones o mujeres heterosexuales, cuya frecuencia osciló entre el 65% y el 75% para América y Europa, esto debido, probablemente, a las altas tasas de coinfección con el HVH-8 que presentan los HSH ^11^. El presente estudio describe una mediana de linfocitos CD4 al momento del diagnóstico de SK de 64 células/µL (RIC: 33-185), comparable a lo descrito en otros continentes; no obstante, se observa una mediana más alta en países con ingresos más altos, como Norteamérica y Europa, 87 células/µL (RIC: 20-280), 180 células/µL (RIC: 60-348), respectivamente ^4^. 

A pesar de que el TARV ha aportado grandes beneficios, el SK ahora se asocia con niveles más altos de recuento de linfocitos CD4, incluso en pacientes adherentes al tratamiento ^12^ ^,^ ^13^. Los estudios han documentado este hallazgo sobre las tendencias en el recuento de linfocitos CD4 desde que Maurer *et al*. informaron de un grupo de nueve pacientes con VIH en tratamiento antirretroviral que desarrollaron SK en el contexto de un buen estado inmunológico (CD4 superior a 300 células/µL) y carga viral indetectable ^14^.

En concordancia con ellos, el presente estudio encontró ocho individuos que presentaron recuentos de linfocitos CD4 superiores a 300 células/µL y, de estos, seis con carga viral indetectable. Algunos autores han planteado la hipótesis de que el elevado número de pacientes de edad avanzada podría alterar la integridad del sistema inmune en la infección por VIH de larga duración ^15^. No obstante, Unemori *et al*. en 2013, exploraron los posibles mecanismos de este fenómeno y descubrieron que la pérdida progresiva de la función inmunológica durante la edad avanzada, denominada inmunosenescencia, podría estar directamente asociada con el desarrollo del SK ^16^.

La principal limitación de este estudio fue el sesgo de información, ya que al ser retrospectivo en un periodo de 18 años, la información se limitó a lo disponible en una base de datos y a lo adicionalmente obtenido de la revisión de historias clínicas de pacientes que todavía seguían vivos. Otra limitante fue el sesgo de selección, puesto que se eligieron a todos los pacientes con VIH que desarrollaron SK. Sin embargo, se subestimó el número real de casos, debido a que antes se carecía de técnicas de recolección de datos, por lo que no se pudo determinar la prevalencia o incidencia de esta enfermedad.

En conclusión, las características asociadas al SK encontradas en nuestro medio fueron similares a las descritas en la literatura. Es predominantemente de varones jóvenes, con orientación sexual hacia HSH, quienes presentan recuentos bajos de linfocitos CD4, y con compromiso cutáneo como presentación más frecuente, pero al menos la mitad de ellos, con compromiso visceral asociado. Asimismo, es importante resaltar que, en esta serie, como en otras descritas recientemente, también se identificaron pacientes con SK con recuentos de linfocitos CD4 superiores a 300 células/µL.
